# Plastid genomes of two brown algae, *Ectocarpus siliculosus *and *Fucus vesiculosus*: further insights on the evolution of red-algal derived plastids

**DOI:** 10.1186/1471-2148-9-253

**Published:** 2009-10-16

**Authors:** Gildas Le Corguillé, Gareth Pearson, Marta Valente, Carla Viegas, Bernhard Gschloessl, Erwan Corre, Xavier Bailly, Akira F Peters, Claire Jubin, Benoit Vacherie, J Mark Cock, Catherine Leblanc

**Affiliations:** 1 CNRS, FR2424, Computer and Genomics Resource Centre, Station Biologique, Roscoff, France; 2 UPMC Univ. Paris 06, FR2424, Computer and Genomics Resource Centre, Station Biologique, Roscoff, France; 3 Centre of Marine Sciences, University of Algarve, Marine Ecology and Evolution, Faro, Portugal; 4 CNRS, UMR7139, Marine Plants and Biomolecules, Station Biologique, Roscoff, France; 5 UPMC Univ. Paris 06, UMR7139, Marine Plants and Biomolecules, Station Biologique, Roscoff, France; 6 CEA, DSV, Institut de Génomique, Genoscope, Evry, France; 7 CNRS, UMR 8030, Evry, France; 8 Université d'Evry, Evry, France

## Abstract

**Background:**

Heterokont algae, together with cryptophytes, haptophytes and some alveolates, possess red-algal derived plastids. The chromalveolate hypothesis proposes that the red-algal derived plastids of all four groups have a monophyletic origin resulting from a single secondary endosymbiotic event. However, due to incongruence between nuclear and plastid phylogenies, this controversial hypothesis remains under debate. Large-scale genomic analyses have shown to be a powerful tool for phylogenetic reconstruction but insufficient sequence data have been available for red-algal derived plastid genomes.

**Results:**

The chloroplast genomes of two brown algae, *Ectocarpus siliculosus *and *Fucus vesiculosus*, have been fully sequenced. These species represent two distinct orders of the Phaeophyceae, which is a major group within the heterokont lineage. The sizes of the circular plastid genomes are 139,954 and 124,986 base pairs, respectively, the size difference being due principally to the presence of longer inverted repeat and intergenic regions in *E. siliculosus*. Gene contents of the two plastids are similar with 139-148 protein-coding genes, 28-31 tRNA genes, and 3 ribosomal RNA genes. The two genomes also exhibit very similar rearrangements compared to other sequenced plastid genomes. The tRNA-Leu gene of *E. siliculosus *lacks an intron, in contrast to the *F. vesiculosus *and other heterokont plastid homologues, suggesting its recent loss in the Ectocarpales. Most of the brown algal plastid genes are shared with other red-algal derived plastid genomes, but a few are absent from raphidophyte or diatom plastid genomes. One of these regions is most similar to an apicomplexan nuclear sequence. The phylogenetic relationship between heterokonts, cryptophytes and haptophytes (collectively referred to as chromists) plastids was investigated using several datasets of concatenated proteins from two cyanobacterial genomes and 18 plastid genomes, including most of the available red algal and chromist plastid genomes.

**Conclusion:**

The phylogenetic studies using concatenated plastid proteins still do not resolve the question of the monophyly of all chromist plastids. However, these results support both the monophyly of heterokont plastids and that of cryptophyte and haptophyte plastids, in agreement with nuclear phylogenies.

## Background

The endosymbiotic captures of free-living prokaryotes, leading to the evolution of two types of organelles, mitochondria and plastids, are considered to be key events in the establishment and success of extant eukaryotic lineages [1,2]. If all mitochondria are likely to be derived from an α-proteobacterium-like ancestor, possibly due to a single and ancient endosymbiotic event, the history of plastid acquisition in the diverse photosynthetic eukaryotic lineages seems to be more complex [3-6]. It is now largely accepted that a single primary endosymbiotic event involving the capture of a cyanobacterium led to an ancestral primary plastid, which subsequently gave rise to the green plastids of the terrestrial plants and chlorophytes, the rhodoplasts of red algae and the cyanelles of the glaucophytes. Once established, primary red or green algal plastids later spread independently to other eukaryote lineages via secondary or tertiary endosymbioses, whereby a photosynthetic eukaryote was engulfed by another eukaryote. Subsequently, plastids have also been independently lost and/or replaced in several eukaryote lineages, making the reconstruction of plastid evolution very difficult.

The current consensus of eukaryote phylogeny recognizes six putative super-clusters: Opisthokonta, Amoebozoa, Plantae, Chromalveolata, Rhizaria, and Excavata [7,8], but this division is still debated [9,10]. The three primary plastid-containing lineages, Viridiplantae, Rhodophyta and Glaucophyta form the "Plantae" or "Archaeplastida" supergroup. Photosynthetic eukaryotes with secondary or tertiary plastids have evolved independently in the Chromalveolata, Rhizaria, and Excavata [3,5]. Among the secondary plastids, chlorophyll c-containing plastids have been shown to be derived from an ancestral red alga via a secondary endosymbiotic process that took place around one billion years ago [11,12]. This type of plastid is found in Cryptophyta, Haptophyta, Heterokonta (also called stramenopiles) and Dinophyceae algae [3,4]. Cryptophyta, Haptophyta and Heterokonta eukaryotic lineages have been grouped under the name of "Chromista" by Cavalier-Smith [13], and were later associated with the Alveolata, which includes the apicomplexans, dinoflagellates and ciliates, to form the "Chromalveolata" supergroup. In 1999, Cavalier-Smith proposed that all the chlorophyll c-containing plastids were derived from a single secondary endosymbiotic event and that the common ancestor of chromalveolates was originally photosynthetic [14]. During diversification of the four extant chromalveolates lineages, photosynthetic capacity and/or the plastid organelle would then have been independently lost several times in different eukaryotic lineages, such as oomycetes (non-photosynthetic heterokonts), apicomplexa or ciliates (non-photosynthetic alveolates). According to this so-called "chromalveolate" hypothesis, plastid and nuclear genomes have similar evolutionary histories and one would expect monophyly of chromalveolate lineages in both nuclear and plastid phylogenies. This hypothesis has been extensively debated over the last ten years (for recent references, [5,6,15-17]), in part because of incongruence between plastid and nuclear phylogenies [9].

At the nuclear level, both the monophyly of heterokonts and alveolates and that of cryptophytes and haptophytes have received increasing support in recent years (for recent review and references therein, [6]). Two contemporary phylogenetic analyses based on expressed sequences tag surveys of the cryptomonad *Guillardia theta *and the haptophyte *Emiliania huxleyi *supported the close relationship of cryptophyte and haptophyte host lineages [18,19]. In nuclear phylogenies alveolates and heterokonts often form a sister group [9,20]. Unexpectedly, several large scale nuclear phylogenies have also shown a very robust relationship between members of Rhizaria, cercozoans, and these two main clades of the "chromalveolates", but with the exclusion of haptophytes and cryptophytes [18,21,22]. The debate is becoming more complex with the emergence of this new putative SAR (stramenopiles/alveolata/rhizaria) supergroup, as proposed by Burki [23]. Recent phylogenetic studies employing large gene- and taxon-rich datasets continue to question the reality of the "chromalveolate" supergroup, by placing the haptophyte-cryptophyte clade as a sister group to the Plantae [24,25] or by having them emerging independently and separately from the SAR supergroup [10]. It is however well known that reconstructing the evolution of host cell lineages can be difficult, especially because of the chimeric nature of nuclear genomes and because large-scale horizontal gene transfers have occurred in some lineages during evolution [26].

Plastid genomes are less affected by horizontal gene transfer, with some rare exceptions [27]. At the plastid level, the monophyly of chromist plastids is supported by analyses of single genes [28], of small numbers of concatenated plastid genes [12,29], and of larger datasets of plastid-associated genes, i.e. plastid and nuclear-encoded plastid-targeted genes [30-35]. The relationships among chlorophyll c-containing plastids are, however, particularly hard to resolve and the results obtained are sometimes incongruent with host cell phylogenies [9]. Haptophyte plastid genes more often group with the heterokont/dinoflagellate clade, than with those of cryptophytes [30,31,33,34]. A clade grouping haptophyte and cryptophyte species has been inferred from some plastid gene phylogenies [31,33-35]. This clustering was not strongly supported and was highly dependent on the plastid gene dataset used [31,35] and/or on taxon-sampling [33,34]. Other variant topologies have included the placing of dinoflagellates either as a sister-group to haptophyte plastids [30,33] or to heterokont plastids [34,35]. However, a close evolutionary relationship between haptophyte and cryptophyte plastids would be consistent with the presence of a unique laterally transferred bacterial *rpl36 *gene in both plastid genomes [27]. Other multigene analyses produced alternative results, such as low support for the chromist clade [29] or paraphyly of red-algal derived plastids [35,36].

The inability to recover congruencies between plastid and nuclear phylogenies, especially concerning haptophyte and cryptophyte monophyly, may be explained by poor taxon sampling of red algal and chromist species [31,36]. Until now, insufficient sequence data have been available for the chromalveolates, in terms of both nuclear and plastid genome sequences. In public databases, more than 110 complete plastid genomes are available from land plants and green algae, whereas less than 15 sequences belong to red algae or photosynthetic chromalveolate species. Only five complete plastid sequences have been reported for red algal species [36-39]. For the chromalveolates, with the exception of the highly diverged red-algal derived plastid genomes of non-photosynthetic apicomplexans [40] and those of dinoflagellates [41,42], complete plastid sequences have been published for two cryptomonads, *Guillardia theta *and *Rhodomonas salina *[11,31], one haptophyte, *Emiliania huxleyi *[43], 3 diatoms, *Odontella sinensis*, *Phaeodactylum tricornutum *and *Thalassiosira pseudonana *[44,45], one raphidophyte *Heterosigma akashiwo *[46] and one xanthophyte *Vaucheria litorea *[47].

Here we report the complete sequences of the plastid genomes of *Ectocarpus siliculosus *and *Fucus vesiculosus*. These sequences represent the first fully characterized plastid genomes from two distinct orders of Phaeophyceae, namely Ectocarpales and Fucales [48]. We have performed phylogenetic studies using large sets of genes and different reconstruction methods. The results still do not resolve the question of the monophyly of chromist plastids. However the topologies of concatenated plastid protein phylogenetic trees support both the monophyly of heterokont plastids and that of cryptophyte and haptophyte plastids, in agreement with nuclear phylogenies.

## Results

### Structure and gene content of the phaeophyte plastid genomes

**Figure 1:**
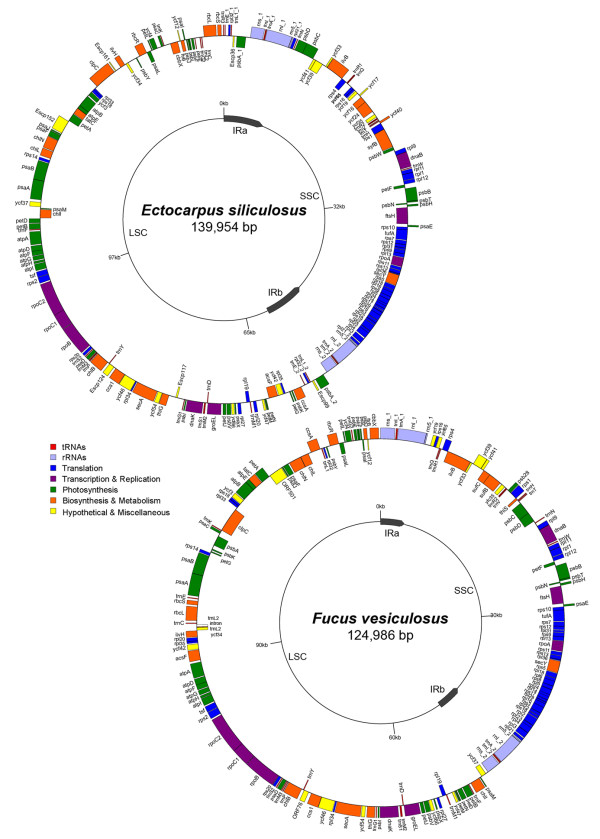
**Plastid genome maps of *E. siliculosus *and *F. vesiculosus***. Genes on the outside of the circles are transcribed clockwise, whereas those on the inside counter clockwise. Annotated genes are colored according to the functional categories shown in the legend and the tRNA genes are indicated by the single-letter code of the corresponding amino-acid. Abbreviations: IR, inverted repeats; SSC, small single-copy region; LSC, large single-copy region.

**Figure 2:**
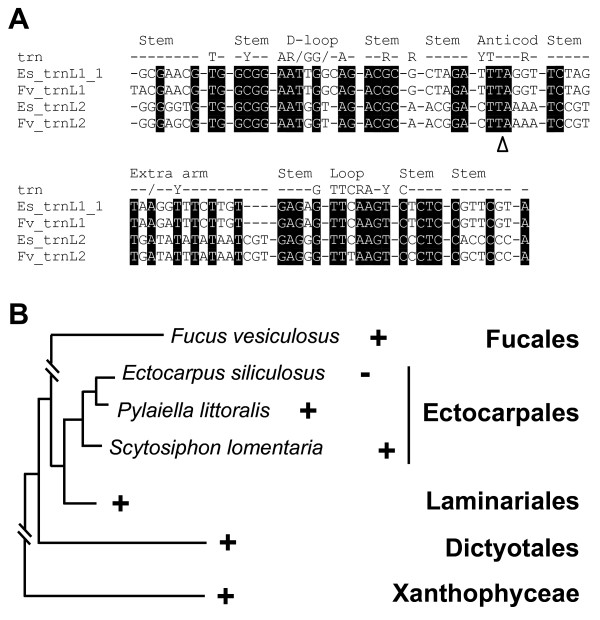
**The canonical group I intron in the plastid tRNA-Leu (*trnL*) gene**. (A) Multiple alignments of plastid *trnL *genes from *E. siliculosus *(Es) and *F. vesiculosus *(Fv), showing the position of the group I intron located in the *F. vesiculosus trnL2 *gene. Structural features of tRNA are indicated on the two first lines. (B) Schematic phylogeny of the phaeophytes (redrawn from [48]), showing the presence (+) and absence (-) of the *trnL *intron in the different orders.

The plastid genomes of *E. siliculosus *and *F. vesiculosus *are 139,954 and 124,986 base pairs (bp) in size, respectively, and both contain two inverted repeat regions (IR). These IRs divide the circular molecules into large (LSC) and small single copy (SSC) regions (Figure 1 and see general features of the two plastid genomes in additional file 1, Table S1). The size difference between the genomes was partly due to the presence of longer IRs of 8,615 bp in the *E. siliculosus *cpDNA. The 4,863 bp *F. vesiculosus *IRs contain only the ribosomal RNA operons. Another reason for the difference in size between the two genomes is the presence of longer intergenic regions in the *E. siliculosus *cpDNA. These sequences represent about 20% of the genome, whereas only 14.5% of the *F. vesiculosus *cpDNA is intergenic. The overall GC content is 30.7% for *E. siliculosus *and 28.9% for *F. vesiculosus*. In both *Fucus *and *Ectocarpus*, the cpDNA IRs contain two ribosomal operons encoding 16S, 23S and 5S rRNA. The *F. vesiculosus *and *E. siliculosus *plastid genomes are predicted to encode a total of 139 and 144 protein-coding genes, and 26 and 27 tRNA genes, respectively, when the duplicated genes in the IRs are counted only once. An intron was identified in the *F. vesiculosus trnL2 *gene, which encodes tRNA-Leu. Interestingly, its closest homologue in *E. siliculosus *cpDNA (93% nucleotide identity) does not possess an intron. The other tRNA-Leu genes in these plastid genomes, *trnL1_1 *of *E. siliculosus *and *trnL1 *of *F. vesiculosus*, present 98% sequence identity to each other and also lack the intron (Figure 2).

Gene organisation is highly similar between the two genomes and around two thirds of both molecules are conserved with respect to both gene identity and order. About 50% of each genome is incorporated into two large, locally collinear blocks. One block contains a large proportion of ribosomal protein-coding genes and covers up to 24% of the plastid genomes. The second block extends between *trnM *and *atpA *and covers 26-27.5% of each genome (Figure 1 and see the MAUVE analysis, provided in additional file 1, Figure S1). When compared to other heterokont plastid genomes, the number of genome rearrangements since the common ancestor of *E. siliculosus *and *F. vesiculosus *is comparable to the number of rearrangements that have occurred since the divergence of the three diatom species (see the reversal distance matrix provided in additional file 1, Table S2). This number increases more than twofold when higher taxonomic levels are considered (e.g., xanthophyte, raphidophyte or diatoms vs. brown algae).

The two plastid genomes are also very similar in terms of total gene content (Table 1). As already found in most of the green and red photosynthetic plastid genomes, excluding those of dinoflagellates [43], they possess the common core set of 44 genes, but with the exception of the *psbZ *gene (listed in additional file 2, Table S3). They also contain 42 additional protein-coding genes, which are only found in red algal and chromist plastid genomes, giving a total of 86 genes that are shared with the red plastid lineage (Table 1). These genes mainly encode essential plastid proteins, involved in transcription, protein synthesis and transport, and photosynthetic metabolism, such as components of ATP synthase, cytochrome, photosystem I and II complexes. Nine genes are shared by all the chromist plastid genomes, but not with all the red algal plastid genomes (Table 1). Another 27 genes are encoded by most heterokont plastid genomes, but are not consistently present in the plastid genomes of haptophytes, cryptophytes and red algae. Of the 17 remaining genes that are common to *E. siliculosus*, *F. vesiculosus *and *V. litorea *cpDNAs, nine are present in the raphidophyte plastid genome, but all are absent from the diatom cpDNAs (Table 1).

**Table 1:** Gene content comparisons between plastid genomes.

Genes		Species													
	**No**.	**Heterokonts**							**Hapto**	**Crypto**		**Rhodophytes**			
					
		**Esil**	**Fves**	**Vlit**	**Haka**	**Ptri**	**Osin**	**Tpse**	**Ehux**	**Gthe**	**Rsal**	**Gten**	**Ppur**	**Ccal**	**Cmer**

83 genes listed in Table S3*, *rpl36*, *rbcR*, *ccsA*	86	+	+	+	+	+	+	+	+	+	+	+	+	+	+
*rpoC1, rpoC2, ycf35, ycf46*	4	+	+	+/-	+	+	+	+	+	+	+	+	+	+/-	-
*cbbX, ccs1, tatC*	3	+	+	+	+	+	+	+	+	+	+	+	-	+/-	+
*petL, petM*	2	+	+	+	+	+	+	+	+	+	+	-	+	-	+
*thiG, thisS*	2	+	+	+	+	+	+	+	+	-	-	+	+/-	+	+
*dnaB, ftsH, petF, psaE, psbX, rpl1, rpl4, rpl11, rpl12, rpl13, rpl18, rpl24, rpl29, rpl32, rpl35, rps20, sufC, ycf33*	18	+	+	+	+	+	+	+	-	+	+	+	+	+/-	+
*psbY*	1	+	+	+	+	+	-	+	-	-	+	-	-	-	+
*ycf41, ycf42, ycf66*	3	+	+	+	+/-	+	+/-	+	-	-	-	-	-	-	-
*psbW, secG, tsf*	3	+	+	+/-	-	+	+	+	+/-	+	+	+/-	+/-	+/-	+/-
*ftrB, ilvB, ilvH*	3	+	+	+	+	-	-	-	-	+	+	+	+	+/-	+/-
*ycf65*	1	+	+	+	+	-	-	-	+	-	-	+	-	+	+
*acsF, petJ, rps1, ycf34, ycf54*	5	+	+	+/-	+	-	-	-	-	-	-	+/-	+/-	+/-	+/-
*chlB, chlL, chlN, rpl9*	4	+	+	+	-	-	-	-	-	-	-	+/-	+/-	+/-	+/-
*ycf19, ycf37*	2	+	+	+	-	-	-	-	+/-	+	+	+	+	+	+/-
*Escp124 (FvORF76) ~ ycf60*	1	+	+	+	-	-	-	-	+	-	-	+	-	+	+
*Escp152 (FvORF501)*	1	+	+	-	-	-	-	-	-	-	-	-	-	-	-
*hlip ~ ycf17*	1	+	-	+	-	-	-	-	-	+	+	-	+	+	+
*syfB*	1	+	-	-	-	+	-	-	-	-	-	+	+	-	-
*Escp36, Escp117, Escp161*	3	+	-	-	-	-	-	-	-	-	-	-	-	-	-

*E. siliculosus *(Esil) and *F. vesiculosus *(Fves) cpDNAs were compared with those of other heterokonts, haptophyte (Hapto), cryptophytes (Crypto) and rhodophytes. Presence (+), absence (-) or presence of only a subset (+/-) of the genes listed in the first column is indicated. Species abbreviations: *Vaucheria litorea *(Vlit), *Heterosigma akashiwo *(Haka), *Phaeodactylum tricornutum *(Ptri), *Odontella sinensis *(Osin), *Thalassiosira pseudonana *(Tpeu), *Emiliania huxleyi *(Ehux), *Guillardia theta *(Gthe), *Rhodomonas salina *(Rsal), *Gracilaria tenuistitipata *(Gten), *Porphyra purpurea *(Ppur), *Cyanidium caldarium *(Ccal), *Cyanidioschyzon merolae *(Cmer).

(*) see additional file 2.

Among the unknown plastid proteins, the conserved open reading frames (ORFs) *Ectocarpus Escp124 *and *Fucus *ORF76 encode putative proteins of 222 and 229 amino-acids, with 48% identity between species. Both protein sequences are predicted to possess five transmembrane helices. A homolog of these plastid proteins is also encoded by the plastid genome of the xanthophyte *V. litorea*. Interestingly, the most similar protein in the public databases is a nuclear-encoded protein, Tic20, found in several apicomplexa species, including *Toxoplasma *and *Plasmodium*. The C-terminal ends of these proteins also share weak similarity with the conserved hypothetical plastid proteins encoded by the *ycf60 *genes of plastid genomes from *E. huxleyi*, *G. tenuistitipata *and Cyanidiales (see partial multiple alignment provided in additional file 3, Figure S2).

### Phylogenetic analyses

**Figure 3:**
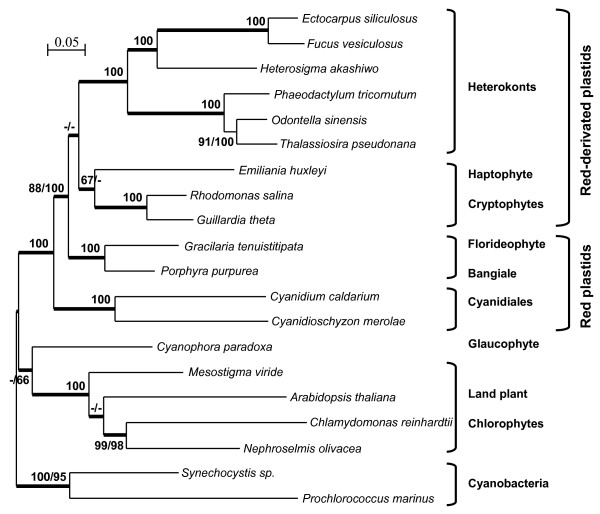
**Maximum likelihood tree constructed from a dataset of 44 concatenated proteins from 20 plastid or cyanobacterial complete genomes**. PHYML and Neighbour Joining trees were constructed based on 8,652 amino-acid sites using cpREV and JTT matrices, respectively. When above 65% and different, bootstrap values (1000 replicates) are provided for PHYML (first value) and NJ (second value) analyses. The thick branches represent ≥ 0.9 posterior probability for Bayesian inference analysis.

For phylogenetic analyses, three concatenated amino acid datasets were constructed (see additional file 2, Table S3) and analysed using maximum likelihood (ML), neighbour joining (NJ) and Bayesian inference (BI) methods. For the ML analyses, cpREV and JTT amino acid substitution matrices gave the same tree topologies (data not shown). Trees were constructed using a dataset of 44 proteins (8,652 amino-acid positions) from a broad range of species, including 13 taxa of red-algal type plastids, 4 taxa of Viriplantae, the glaucophyte *Cyanophora*, and two cyanobacteria (see additional file 2, Table S4 for species list). Plastid sequences of chlorophyll-c-containing dinoflagellates were not included in the analyses because this would have resulted in a significantly reduced common protein dataset. All but four of the nodes in the trees were well resolved and supported by the three different methods (Figure 3). As observed in previous studies, the red-algal and red-derived type plastid sequences grouped together, whereas green plastids formed a separate monophyletic group, derived from the cyanobacterial sequences. In all our analyses, the glaucophyte plastid from *Cyanophora *emerged at the base of the green plastids, with high confidence in the BI analysis but with low bootstrap support in the ML and NJ analyses (56 and 66%). Among the green plastids, the order of branching of *Mesostigma *and *Arabidopsis *was not fully resolved, but the phylogenetic position of *Mesostigma *within the Streptophyta has been studied recently, with expanded taxon sampling of the Viridiplantae [49]. In the other part of the tree, the Cyanidiales grouped together outside a strongly supported clade that includes the Florideophyceae and Bangiophyceae, together with the heterokont, the haptophyte and the cryptophyte plastids. The trees also strongly grouped all heterokont plastids together, with a split between diatom plastid sequences and those of the raphidophyte and phaeophytes. The Florideophyceae and Bangiophyceae branched together with high confidence using all the methods, as did the two species of cryptophytes. In these phylogenetic studies, the haptophyte *E. huxleyi *emerged as the closest branch to cryptophytes in the BI analysis but this topology had low bootstrap support in the ML analysis (67%), and no support in the NJ analysis. The order of branching of the following three major groups: heterokonts, (florideophyte+bangiophyte), and (cryptophytes+haptophyte), was also uncertain. In fact, the clade of heterokonts and (cryptophyte+haptophyte) plastids was only well-supported by the BI analysis, and very poorly (49%) or not supported by the ML and NJ analyses, respectively.

**Figure 4:**
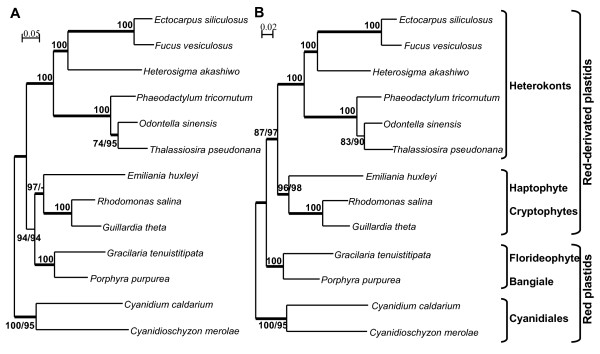
**Maximum likelihood trees constructed from two datasets of concatenated proteins from 13 completed plastid genomes of red algal and chromist species**. A) The full dataset of 83 proteins (16,738 amino-acid sites), and B) the 33 slow-evolving proteins dataset (8,404 amino-acid sites) were used for PHYML and Neighbour Joining analyses, using cpREV and JTT matrices, respectively. When above 65% and different, bootstrap values (1000 replicates) are provided for PHYML (first value) and NJ (second value) analyses. The thick branches represent ≥ 0.9 posterior probability for Bayesian inference analysis.

To strengthen the topology of branching in the region of the tree corresponding to the red-alga derived plastids, we decided to increase the protein dataset by focusing the phylogenetic studies on 13 species. A full dataset of 83 plastid-encoded proteins (16,738 amino acid positions) was analyzed in parallel with a sub-dataset of 33 slowly-evolving plastid proteins, excluding the fast-evolving proteins (Figure 4). Using the PhyloBayes software, the values of the saturation index have been calculated for each dataset. The observed and predicted homoplasy rates are, respectively, 1.98 ± 0.05 and 2.00 ± 0.05 for the 83-protein dataset, and 1.01 ± 0.03 and 1.00 ± 0.04 for the 33-protein dataset. These results show that the exclusion of the fast-evolving proteins tends to decrease the global level of saturation. Both trees still showed two well-supported plastid groups, corresponding to heterokonts and the Cyanidiales. Globally, the branches that were strongly supported by the 44-protein dataset were maintained. Interestingly, the group formed by haptophyte and cryptophyte plastids had greater support in the ML analysis (97% bootstrap value) but little support with NJ method with the 83-protein dataset (Figure 4A) and was strongly supported by the three methods in the analyses of the slowly-evolving proteins (Figure 4B). Compared to the 44-protein trees, the 83- and 33-protein trees differed in their branching patterns with respect to the (florideophyte+bangiophyte) and the (cryptophytes+haptophyte). Both the ML and NJ trees built with the dataset of 83 proteins clustered these two groups with high bootstrap values, whereas the red algal plastids were found outside the clade of heterokont/(cryptophyte+haptophyte) plastids in the 33-protein trees. This latter topology was strongly supported in the ML, NJ and BI analyses (Figure 4B).

**Figure 5:**
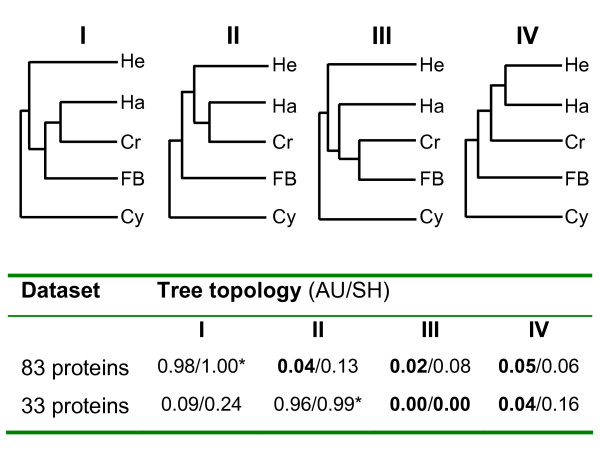
**Likelihood AU and SH tests of four alternative tree topologies, using the two different datasets**. In tree topologies, the abbreviations used are: He, Heterokont plastids; Ha, Haptophyte plastids; Cr, Cryptophyte plastids; FB, Florideophyte+Bangiophyte plastids; Cy, Cyanidiales plastids. In the table, the best tree is indicated by a star. Boldface type corresponds to *P *< 0.05.

To further test these phylogenetic positions, we compared different topologies by performing the approximately unbiased (AU) and Shimodaira-Hasegawa (SH) tests (Figure 5). Four topologies were selected to evaluate two hypotheses: 1) Are chromist plastids indeed monophyletic; 2) Are haptophyte plastids specifically related to cryptophyte plastids to the exclusion of heterokont or (florideophyte+bangiophyte) plastids? Our analyses showed that, for the 83- and 33-protein datasets, the best topologies correspond to the trees shown in Figure 4A (topology I) and 4B (topology II), respectively. Considering the two datasets, these two topologies had a much higher likelihood in AU and SH tests, than topologies that place either the haptophyte plastid outside a (cryptophyte+(florideophyte+bangiophyte)) clade (topology III) or that propose that the closest relationship is between heterokont and haptophyte plastids (topology IV). For the 83-protein dataset, the three topologies (II, III and IV) were significantly rejected with p value under 0.05 for AU tests, but not for SH tests. For the 33-protein dataset, the topology I could not be significantly rejected by both tests (*P *= 0.09; *P *= 0.24), whereas the other topologies were refuted with *P *values below the significance level.

## Discussion

### Monophyly and evolution of heterokont plastid genomes

Until very recently, all of the plastid genomes available for the heterokont lineage were from diatoms (*O. sinensis*, *P. tricornutum *and *T. pseudonana*), and these genomes featured conserved gene content and gene clusters [45]. Along with the recently published plastid genomes of two strains of the raphidophyte *H. akashiwo *[46]) and the xanthophyte *V. litorea *[47], the complete sequences of the *E. siliculosus *and *F. vesiculosus *plastid genomes presented here significantly increase the number and diversity of heterokont plastid genomes available, allowing a more extensive comparison of these genomes. Our results support a unique origin for all heterokont plastids, based on similarity in terms of gene content (Table 1) and on their forming a strongly supported group in all our phylogenetic analyses (Figures 3 and 4). These analyses were, therefore, consistent with the well established monophyletic origin of the heterokont host cell [10,21,23]. However, despite their common origin, genome comparisons revealed specific traits in the evolution of heterokont plastids during the diversification of the different heterokont orders.

All the Xanthophyceae or Phaeophyceae plastid genomes analyzed to date, including that of *F. vesiculosus *described here, contain a tRNA-Leu gene with a single intron [47,50]. This canonical group I intron is thought to have been acquired from the ancestral cyanobacterial endosymbiont and to have been lost independently in several lineages of plastids, including the red algae and almost all their secondary plastid derivatives, except the Xanthophyceae/Phaeophyceae lineage [50]. Given the high sequence similarities found between these plastid tRNA-Leu genes in *V. litorea*, *F. vesiculosus *and *E. siliculosus *(86 to 93% sequence identity), they are probably derived from the same ancestral tRNA-Leu gene, containing the endosymbiotic derived intron. In the *E. siliculosus *gene, its loss is likely to be recent because it is still present in the plastid tRNA-Leu genes of Laminariales species and of two Ectocarpales, *Pylaiella littoralis *and *Scytosiphon lomentaria *(Figure 2) [50]. This feature is evidence for continued evolution of brown algal plastid genomes within the recently-derived order Ectocarpales [48,51].

In terms of gene content, the brown algal plastid genomes seem to be more closely related to those of *V. litorea *and of *H. akashiwo *than to those of diatoms and this is consistent with evolutionary relationships of the nuclear compartment [51,52]. Although the structural organisation of plastid genomes is highly conserved within the brown algae (additional file 1, Figure S1) and within diatoms [45], there is evidence of intensive gene rearrangements having occurred earlier in evolution after the separation of diatoms from raphidophytes, xanthophytes and phaeophytes. Moreover, more extensive gene losses seem to have occurred in diatom plastid genomes than in other heterokonts (Table 1). These genes could have been transferred to the nucleus or replaced by bacterial counterparts, functionally-integrated through horizontal gene transfer as often seen in the diatom nuclear genome [53]. All these data, together with the topologies of plastid phylogenetic trees (Figure 3 and 4) support a relatively ancient split between diatoms and the raphidophyte-phaeophyte clade, in agreement with the early divergence of the Bacillariophyceae from the other photosynthetic heterokont lineages in nuclear phylogenies [51,52].

### What is the closest relative of the heterokont plastid clade?

A critical step for the transformation of the endosymbiont into a permanent organelle was the establishment of an efficient protein targeting and translocation system from the nucleus to the plastid [1,4]. The canonical Tic/Toc protein import complex of secondary plastids was inherited from the first red-algal endosymbiont, with components of both eukaryotic and eubacterial origin [1,54,55]. Both brown algal plastid genomes have a gene (*Escp124 *in *Ectocarpus *and *ORF76 *in *Fucus*) that shares similarity with the Tic20-like genes in xanthophyte, haptophyte and red algal plastid genomes. There are no homologues of this gene in raphidophyte, diatom and cryptophyte plastid genomes (Table 1). This plastid-encoded Tic20 gene (also called *ycf60*) encodes a small membrane protein and is thought to be endosymbiont-derived with a cyanobacterial origin [1,54,55]. Interestingly, the highest similarity scores of brown algal and xanthophyte plastid ORFs were found with a homologous protein encoded in the nucleus of several apicomplexan species, including *Toxoplasma *and *Plasmodium*. In *T. gondii*, this Tic20-like protein has been shown to be essential for protein import into the apicoplast [56] and is therefore likely to be linked to apicoplast evolution [4]. Escp124 and ORF76 protein sequences are also predicted to have five transmembrane regions, suggesting a putative location in the plastid membrane. It is now widely accepted that alveolates and heterokonts are derived from a common host cell ancestor. *Escp124 *and *ORF76 *could be footprints of a common photosynthetic ancestor of heterokonts and apicomplexans. This hypothesis is in agreement with several recently published studies suggesting that contemporary alveolates are derived from a photosynthetic ancestor. These studies include the characterization of a photosynthetic alveolate closely related to apicomplexan parasites [57], the identification of plastid-derived genes in a non-photosynthetic alveolate [58] and the identification of remnant algal-related genes in ciliates [59].

### Is the monophyly of chromist plastids still in doubt?

All the phylogenetic analyses carried out in this study suggest that the red algal ancestor of chromist plastids was more closely related to the more recently evolved red algae (Florideophyceae and Bangiophyceae) than to Cyanidiales, confirming the report by Sanchez-Puerta et al. [33]. It is worth mentioning that Cyanidiales are extremophile unicellular red algae and have been shown to be the earliest diverging red algal group. They emerge very distinctly from the other multi-cellular red algal taxa in nuclear phylogenies [29]. Within the chromist plastid clade, most plastid phylogenies have hitherto featured a clade grouping haptophyte and heterokont plastids [29,30] and the relationship between haptophyte and cryptophyte plastids was never strongly recovered in previous studies [31,33-35]. These conflicting results have been discussed in the light of taxon- or data-sampling limitations [31,34]. Our results do not support a preferential link between heterokont and haptophyte plastids, neither in terms of gene content (Table 1) nor phylogenetic relationship. Moreover, these phylogenetic analyses strongly support the monophyly of haptophyte and cryptophyte plastids (Figure 4). In general, addition of taxa has been shown to reduce support for previously robust clades, whereas the addition of more positions has been shown to increase support regardless of the topology [60]. Indeed this topology has high confidence, especially when the dataset of genes was increased or slowly-evolving proteins were selected. Moreover, whatever the datasets used, with or without fast-evolving proteins, AU tests significantly rejected topologies separating haptophyte and cryptophyte plastids. The monophyly of haptophyte and cryptophyte plastids is in complete agreement with recent nuclear phylogenies that support a common origin of their host cells [18,19] and with a previous study that identified a unique, laterally transferred bacterial gene in plastid genomes from these two groups [27].

Horizontal gene transfers into plastid genomes happened only rarely after the establishment of the endosymbiont within the host cell. The major events which can affect the structure of the organelle genome are gene transfer to the nucleus and/or gene loss. Indeed, red algal plastid genomes possess more than 230 protein-coding genes while those derived from a red-algal endosymbiont encode less than 150, of which more than half are shared by all the genomes (Table 1). An exceptional case is the drastic reduction of plastid minicircular genomes of peridinean dinoflagellates [41]. In other plastid genomes derived from a red algal endosymbiont, the remaining pool of genes is the result of losses that have occurred independently in the different lineages and of retention that could constitute interesting fingerprints of ancestral plastid gene contents. A comparison of gene content did not reveal any particular relationships between heterokonts and cryptophytes/haptophytes and therefore did not provide support for a common history. For the phylogenetic analyses, whereas the use of the complete dataset supported a different red-algal origin for heterokont plastids (Figure 4A), monophyly of all chromist plastids was recovered when the most conservative data was used in the phylogenetic reconstruction (Figure 4B), as previously observed [33,36]. Other studies have also shown the disruption of the monophyly of chromist plastids [31,33,35]. Our dataset and taxa sampling are not sufficient to completely refute or confirm the polyphyly of chromist plastids, given that the monophyletic topology does not significantly exclude the polyphyletic one when using the slow-evolving proteins (Figure 5). The slowly evolving proteins may reflect more ancient divergences, but the exclusion of fast-evolving proteins decreases the number of analysed amino-acid positions by a factor of two and the issue of dataset size is critical in plastid multi-gene phylogenetic studies [34]. In the context of the chromalveolate hypothesis, the major separation between cryptophyte/haptophyte and heterokont/alveolate host cells is more likely to have occurred very early after the secondary endosymbiosis. An alternative origin of heterokont/alveolate plastids has recently been proposed, with laterally transferred red-algal derived plastids from the haptophyte/cryptophyte clade into the heterokont/alveolate lineage [5,61]. The monophyly of all chromist plastids is also consistent with this tertiary endosymbiosis hypothesis, if the heterokont plastids were captured before the divergence between the haptophyte and cryptophyte host lineages. It is however clear that plastid phylogenies alone will not resolve these currently discussed questions about vertical or lateral inheritance of red-algal derived plastids [16,17].

It has been shown that plastid metabolism could also involve a significant number of nuclear-encoded proteins recruited from diverse origins, such as laterally transferred genes from Chlamydiae [62] or green algae [63-65]. Phylogenies based on nuclear-encoded plastid-targeted proteins could then trace and reflect complex evolutionary pathways, whereas phylogenies based on complete sets of plastid-encoded genes should better reflect the evolution of the organelle since its engulfment by the host cell. As illustrated by the high resolution of the heterokont plastid clade, additional plastid genomes from haptophytes, cryptophytes and dinoflagellates, but certainly also from other evolved red algae will be required to fully resolve chromist plastid phylogenies and, subsequently, test the different hypotheses concerning red-algal derived plastid origin(s).

## Conclusion

In conclusion, this study of two novel plastid genomes belonging to brown algal species has shown the importance of increased taxon sampling when analysing phylogenetic relationships based on large datasets. As expected, the phylogenetic analyses showed that heterokont plastids are monophyletic, although very diverse in terms of gene arrangement. There is also evidence that some heterokont (phaeophyte and xanthophyte) plastids have retained finger-prints indicating a common ancestory with alveolate plastids. Moreover, monophyly of haptophyte and cryptophytes plastids was strongly recovered whatever the dataset or the method used, in complete agreement with large-scale nuclear phylogenies.

## Methods

### Algal material and DNA extraction

*E. siliculosus *strain Ec32 (CCAP1310/4) was cultivated under laboratory conditions as previously described [66] and total DNA was prepared according to the method of Apt et al. [67].

*F. vesiculosus *was collected from the field (Ria Formosa Natural Park, Portugal) and DNA was extracted from isolated plastids. Briefly, 20 g apical tissue free from visible epiphytes was cleaned by 2 min exposure in bleach (1% in filtered natural seawater), rinsed and homogenized in 100 mL cold extraction buffer containing 0.05 M MES (pH 6.1), 0.5 M sorbitol, 1 mM MgCl_2_, 1 mM MnCl, 0.5 mM K_2_HPO_4_, 5 mM EDTA, 1% BSA, 2% PVP, and 2 mM Na-ascorbate. The homogenate was passed through cotton gauze and 1 μm nylon mesh, centrifuged for 2 min at 2000 × *g *at 4°C. The supernatant was transferred to new 50 mL tubes and centrifuged at 5000 × *g *for 5 min. The pellet containing plastids was gently resuspended in a total of 10 mL of extraction buffer and re-centrifuged (5 min, 5000 × *g*, 4°C). The pellet was resuspended in new extraction buffer and applied to a 30:50% sucrose step gradient. After centrifugation for 45 min at 5000 × g (4°C), the plastids were removed from the 30 and 50% sucrose interface, carefully resuspended in a buffer containing 0.05 M HEPES (pH 7.5), 0.5 M sorbitol, 1 mM MgCl_2_, 1 mM MnCl, 0.5 mM K_2_HPO_4_. After observation under the microscope to determine the quality of the plastid preparation, plastids were centrifuged again for 10 min at 5000 × g. The supernatant was removed and plastids were stored at -80°C prior to DNA extraction using the CTAB method [68].

### Genome Sequencing, Assembly and Annotation

For *E. siliculosus*, several scaffolds corresponding to plastid DNA were detected by similarity to other plastid genomes in an assembly of shotgun sequenced total genomic DNA produced by Genoscope http://www.genoscope.cns.fr/spip/-Ectocarpus-siliculosus-.html. These scaffolds were removed from the rest of the sequence data and the sequence of the circular genome was completed by manual assembly and PCR amplification of gap regions. The plastid genome was annotated using the GenDB interface [69], available through the bioinformatics' facilities of the Marine Genomics Europe Network of Excellence.

For *F. vesiculosus*, two main strategies were used to obtain the full genome sequence: 1) Plastid-enriched DNA (cpDNA) was digested (*Hind*III), and cloned into pBluescript II (SK-) (Stratagene). Positive colonies were randomly picked and those with inserts > 1 Kb after digestion were end-sequenced. 2) Plastid DNA was used to make uncloned, adaptor-ligated libraries for a genome-walking approach using long-distance PCR (GenomeWalker kit, Clontech, Palo Alto, USA). Gaps in the genome were filled by PCR, based on predicted gene organization in red-lineage plastids. The *F. vesiculosus *plastid genome was assembled using CodonCode Aligner (CodonCode Corp., USA). Protein coding genes and putative open reading frames (ORFs) were identified by database comparison (Blastx, [70]) and online tools (ORF Finder, NCBI). Ribosomal and tRNA genes were identified using RNAmmer http://www.cbs.dtu.dk/services/RNAmmer/[71] and ARAGORN http://130.235.46.10/ARAGORN/[72], respectively.

The two plastid sequences are available under the following EMBL accession numbers: *E. siliculosus *(FP102296) and *F. vesiculosus *(FM957154). The physical maps of the circular genome were drawn using GenomeVx (freely available at wolfe.gen.tcd.ie/GenomeVx/).

### Phylogenetic Analyses

For global gene content comparisons, the two brown algal plastid genomes were analysed together with those of the xanthophyte *V. litorea *[47] and the raphidophyte *H. akashiwo *[46] plus the 15 algal sequences and the two reference cyanobacterium genomes analysed by Khan et al. [31]. The phylogenetic analyses were conducted with a total of two cyanobacterium and 18 plastid genomes, including four complete genomes from red algae and nine from chromist species (see additional file 2, Table S4). Three concatenated protein datasets were constructed from these genomes (additional file 2, Table S3). The first dataset corresponded to the 44 plastid protein-coding genes shared by all 20 species. In addition, a larger dataset of 83 proteins was built using all the plastid proteins common to the 13 red, cryptophyte, haptophyte and heterokont algae. A list of gene synonyms used during this study is provided in additional file 2 (Table S5), together with complementary gene annotation information. Single and concatenated protein sequences were aligned using MUSCLE [73] and each alignment was further optimised using GBlocks [74]. Datasets for individual genes were first analysed using maximum likelihood, in order to eliminate genes derived from horizontal transfer. Only the *rpl36 *protein phylogeny suggests a non red-algal origin for the haptophyte and cryptophyte genes, which grouped far outside the red algal and heterokont cluster, as previously reported [27]. This gene was therefore eliminated from the full 83-protein dataset. The average distance was calculated for each protein with Tree-Puzzle [75]. We excluded 50 "fast-evolving" protein sequences to produce a dataset of 33 "slowly-evolving" proteins, which present an average distance under the threshold of 0.6. This value was chosen in order to conserve at least half of the analysed positions for the 33-protein dataset.

Phylogenetic analyses of concatenated protein data were carried out on 8,652, 16,738 and 8,404 amino acids corresponding, respectively, to the 44-, 83- and 33-protein datasets. A Maximum Likelihood (ML) approach was used to reconstruct phylogenetic trees using PHYML [76] under both cpREV [77] and JTT [78] amino acid substitution matrices with 4 gamma-distributed rate categories and estimated invariable sites. The neighbor-joining (NJ) method was performed with JTT amino acid substitution matrix using the Phylip software package [79]. For both the ML and NJ methods, bootstrap analyses of 1,000 replicates were used to provide confidence estimates for the phylogenetic tree topologies. Finally, Bayesian inference (BI) analyses were performed with PhyloBayes 3.1d [80] using 4 gamma-distributed rate categories. PhyloBayes was run using the site-heterogeneous CAT model as described in Lartillot et al. [81] and two independent chains with a total length up to 25,000 cycles, discarding the first 25% as burn-in and calculating the posterior consensus tree. Furthermore, a saturation test was performed on the different datasets to calculate the observed and predicted homoplasy rates as described in the PhyloBayes user manual.

To statistically test the topologies of the trees, approximately unbiased (AU) and Shimodaira-Hasegawa (SH) analyses were performed on four topologies. These were selected to reflect the relative positions of haptophyte, cryptophyte and heterokont plastids and were generated by rearrangement of ML and NJ trees (if required). Site likelihoods for each topology were calculated using Tree-Puzzle on the two different concatenated datasets and the AU/SH tests were performed using CONSEL 0.1 [82].

## Authors' contributions

GLC, BG, CL annotated the *E. siliculosus *cpDNA. GLC, CL carried out the phylogenetic analysis. GP, MV, CV sequenced and assembled the cpDNA of *F. vesiculosus*. GP annotated the *F. vesiculosus *genome. GLC, GP, CL performed the comparative genomic analyses on both plastid genomes. AFP obtained and provided *E. siliculosus *cultures. CJ, BV sequenced and provided plastid contigs of *E. siliculosus*. EC, XB participated in design of phylogenetic and statistical approaches. GLC, GP, JMC contributed to manuscript writing. JMC helped to supervise the project. CL conceived and designed the project, wrote the manuscript. All authors read and approved the final manuscript.

## Supplementary Material

Additional file 1 **Additional data and analyses on the plastid genomes of *E. siliculosus *and *F. vesiculosus***. Tables S1 and S2, general features of the two phaeophyte plastid genomes and reversal distance matrix for pairwise comparisons between heterokont plastid genomes. Figure S1, MAUVE genome comparison between *E. siliculosus *and *F. vesiculosus *plastid genomes. Click here for file

Additional file 2 **Genes and genomes used in the phylogenetic studies**. Tables S3 to S5, lists of genes, accession numbers of plastid and bacterial genomes and complementary information about gene synonyms and about some protein-encoded genes used in the phylogenetic studies. Click here for file

Additional file 3 **Partial multiple alignment of Tic20 and yfc60 protein homologs**. Figure S2 showing partial multiple alignment of Tic20 and yfc60 protein homologs from red alga-derived plastid and apicomplexan genomes. Click here for file
