# Supplementary material for: Plastid genomes of two brown algae, Ectocarpus siliculosus and Fucus vesiculosus: further insights on the evolution of red-algal derived plastids
Source: BMC Evol Biol. 2009 Oct 16;9:253. doi: 10.1186/1471-2148-9-253 (PMC2765969; doi:10.1186/1471-2148-9-253)
Supplement: Additional file 1 — Additional data and analyses on the plastid genomes of E. siliculosus and F. vesiculosus. Tables S1 and S2, general features of the two phaeophyte plastid genomes and reversal distance matrix for pairwise comparisons between heterokont plastid genomes. Figure S1, MAUVE genome comparison between E. siliculosus and F. vesiculosus plastid genomes. [file 1471-2148-9-253-S1.PDF]

Table S1. General features of the two phaeophyte plastid genomes.

	Ectocarpus siliculosus	***Fucus vesiculosus***
Genome length (bp)	139,954	124,986
Inverted repeat (IR)	17,230	9,726
Small single-copy region (SSC)	42,714	40,347
Large single-copy region (LSC)	80,010	74,913
G+C content (%)	30.7	28.9
Predicted coding sequence (%)	80.3	85.5
Protein-coding sequences (%)	72.2	76.6
Total predicted genes (located in the IRs)	185 (22)	173 (10)
Protein-coding genes	148 (8)	139
rRNA genes	6 (6)	6 (6)
tRNA genes	31 (8)	28 (4)
Introns	0	1

Table S2. Reversal distance matrix for pairwise comparisons between available heterokont plastid genomes. Gene order and orientation (+/- strand) for genes present were compared, using *E. siliculosus* as reference. Values in the table are minimum numbers of steps in pairwise comparisons necessary to explain the differences (as rearrangements) between genomes.

**Species**	**Esil**	**Fves**	**Vlit**	**Haka**	**Ptri**	**Tpse**	**Osin**
**Esil**	-	16	30	38	37	36	37
**Fves**		-	30	36	38	39	37
**Vlit**			-	36	37	36	38
**Haka**				-	34	38	34
**Ptri**					-	18	10
**Tpse**						-	16
**Osin**							-

Species abbreviations: *Ectocarpus siliculosus* (Esil), *Fucus vesiculosus* (Fves), *Vaucheria litorea* (Vlit) *Heterosigma akashiwo* (Haka), *Phaeodactylum tricornutum* (Ptri), *Thalassiosira pseudonana* (Tpse), *Odontella sinensis* (Osin). See Table S1 for genome accession numbers.

The genome rearrangement analysis was performed using GRIMM [(*)] (http://nbcr.sdsc.edu/GRIMM/mgr.cgi). A list of homologous genes present in all the taxa was prepared and genes were coded for order and orientation (+/- strand) relative to *E. siliculosus*. The analysis was performed for circular and signed (+/- orientation) genomes for this list of common genes (losses and/or inter-chromosomal transfers, i.e., plastid to nuclear transfer, cannot be incorporated) to give a matrix of reversal distance between genome pairs, i.e., the minimum number of steps required to rearrange genome 1 into genome 2.

[(*)] Tesler G. 2002. GRIMM: genome rearrangements web server. Bioinformatics 18:492-493.

Figure S1.

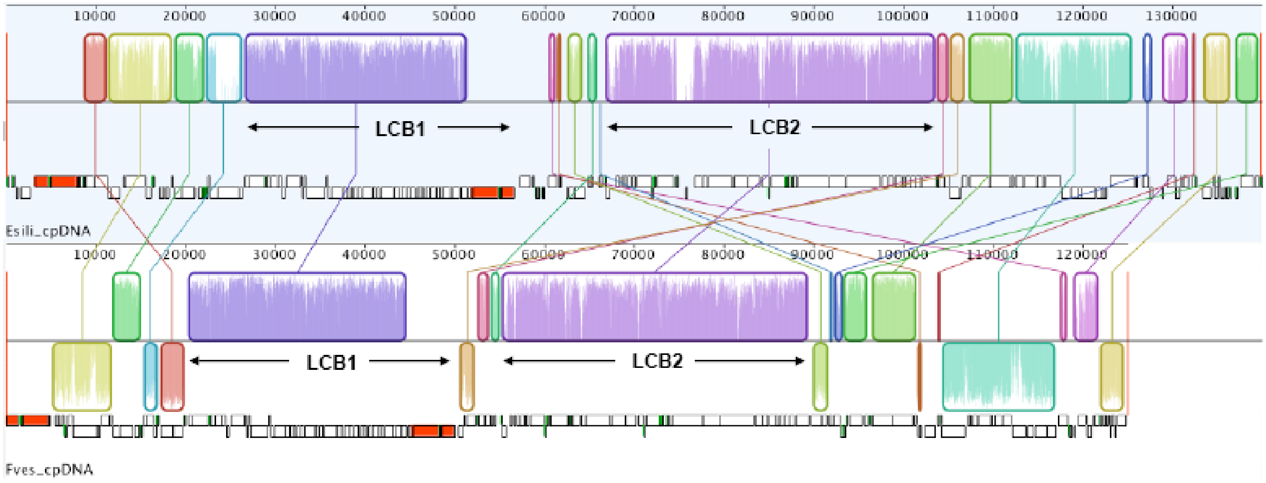

Figure S1. MAUVE genome comparison between *E. siliculosus* (upper, light blue background) and *F. vesiculosus* (lower, white background) plastid genomes.

The (linearized) genomes are represented by small white (protein coding genes) or red (rRNA genes) boxes. Locally collinear blocks (LCBs) identified by MAUVE are colour-coded; links between LCBs are indicated by the thin coloured lines. In the lower portion of the figure, *F. vesiculosus* LCBs lying below the black line have an inverse orientation relative to *E. siliculosus*. The comparison was made using the MauveAligner algorithm with seed weight = 9 and minimum LCB = 50. The MAUVE [(*)] genome alignment software package was implemented in Geneious (4.0.3).

The two major syntenic regions, LCB1 and LCB2 are indicated. LCB1 contains 48 genes, from the *rpl9* to *rns_2* genes. An overlap of 8 bases between *rpl4*/*rpl23* is present in both taxa in LCB1. LCB2 covers 40 (*E. siliculosus*) and 39 genes (*F. vesiculosus*), respectively. The additional gene present in *E. siliculosus*, *Escp117*, encodes a putative protein that has no counterpart in *Fucus*. Numerous smaller regions (from 2 to 13 genes) show strict synteny or identical organisation with an inverted order. Two pairs of same-strand overlapping genes are common to both genomes: *psbC*/*psbD* with 53 overlapping nucleotides (covering the 17 C-terminal codons of *psbD*) and *ycf16*(*sufC*)/*ycf24*(*sufB*) with 4 nucleotides (overlap between the start and stop codons).

[(*)] Darling ACE, Mau B, Blatter FR, Perna NT. 2004. Mauve: multiple alignment of conserved genomic sequence with rearrangements. Genome Res. 14:1394-1403.
