# Supplementary material for: Plastid genomes of two brown algae, Ectocarpus siliculosus and Fucus vesiculosus: further insights on the evolution of red-algal derived plastids
Source: BMC Evol Biol. 2009 Oct 16;9:253. doi: 10.1186/1471-2148-9-253 (PMC2765969; doi:10.1186/1471-2148-9-253)
Supplement: Additional file 2 — Genes and genomes used in the phylogenetic studies. Tables S3 to S5, lists of genes, accession numbers of plastid and bacterial genomes and complementary information about gene synonyms and about some protein-encoded genes used in the phylogenetic studies. [file 1471-2148-9-253-S2.PDF]

Table S3. Gene datasets used in the phylogenetic studies.

Dataset	Genes names
**44-proteins**	atpA, atpB, atpE, atpF, atpH, petA, petB, petD, petG, psaA, psaB, psaC, psaJ, psbA, psbB, psbC, psbD, psbE, psbF, psbH, psbI, psbJ, psbK, psbL, psbN, psbT, rpl14, rpl16, rpl2, rpl20, rpoA, rpoB, rps11, rps12, rps14, rps18, rps19, rps2, rps3, rps4, rps7, rps8, ycf3, ycf4
**83-proteins**	atpA, atpB , atpD, atpE, atpF, atpG, atpH, atpI, chlI, clpC, dnaK, groEL, petA, petB, petD, petG, petN, psaA, psaB, psaC, psaD, psaF, psaI, psaJ, psaL, psaM, psbA, psbB, psbC, psbD, psbE, psbF, psbH, psbI, psbJ, psbK, psbL, psbN, psbT, psbV, rbcL, rbcS, rpl14, rpl16, rpl19, rpl2, rpl20, rpl21, rpl22, rpl23, rpl27, rpl3, rpl31, rpl33, rpl34, rpl5, rpl6, rpoA, rpoB, rps10, rps11, rps12, rps13, rps14, rps16, rps17, rps18, rps19, rps2, rps3, rps4, rps5, rps7, rps8, rps9, secA, secY, sufB, tufA, ycf12, ycf3, ycf39, ycf4
**33-proteins**	atpA, atpB, atpH, chlI, dnaK, groEL, petB, petD, petN, psaA, psaB, psaC, psaD, psbA, psbB, psbC, psbE, psbF, psbH, psbI, psbJ, psbL, psbN, psbT, rbcL, rbcS, rpl14, rpl16, rps12, rps19, sufB, tufA, ycf3

Table S4. Plastid and bacterial genomes used in the global genome comparisons and the phylogenetic studies.

Species	Accession Number	Date
Arabidopsis thaliana	NC_000932	30-JAN-2008
Chlamydomonas reinhardtii	NC_005353	08-FEB-2008
Cyanophora paradoxa	NC_001675	09-OCT-1995
Mesostigma viride	NC_002186	06-AUG-2007
Nephroselmis olivacea	NC_000927	21-FEB-2007
Prochlorococcus marinus	NC_009976	25-JAN-2008
Synechocystis sp.	NC_000911	26-MAR-2008
Cyanidioschyzon merolae *	NC_004799	30-JUL-2007
Cyanidium caldarium *	NC_001840	18-NOV-1999
Ectocarpus siliculosus *	FP102296	*this study*
Emiliania huxleyi *	NC_007288	26-FEB-2007
Fucus vesiculosus *	FM957154	*this study*
Gracilaria tenuistitipata *	NC_006137	13-AUG-2004
Guillardia theta *	NC_000926	15-SEP-1998
Heterosigma akashiwo *	EU168191	05-JUN-2008
Vaucheria litorea °	NC_011600	01-DEC-2008
Odontella sinensis *	NC_001713	21-FEB-2007
Phaeodactylum tricornutum *	NC_008588	13-APR-2007
Porphyra purpurea *	NC_000925	25-APR-1996
Rhodomonas salina *	NC_009573	28-MAR-2008
Thalassiosira pseudonana *	NC_008589	17-APR-2007

(*) Species used in both protein datasets analyses, i.e. the 44-proteins and 83-proteins datasets, whereas the other species appear only in the 44-proteins dataset analyses. (°) Species only used in comparisons of global genome rearrangements and gene contents.

Table S5. Complementary information about gene synonyms used for gene content comparisons between cyanobacterial and plastid genomes, and about protein-encoded genes used in the phylogenetic analyses.

ftsH(ycf25), groEL(groL), petM(ycf31), psbW(psb28), sufB(ycf24), ycf33(Escp43), ycf46(ORF491), petL(ycf7), petN(ycf6), psbZ(ycf9), syfB(pheT), ilvH(ilvN), cbbX(cfxQ), sufC(ycf16), thiS(ycf40), ycf41(Escp41), acsF(ycf59), ycf65(psrp3), atpE(atpC), rpl1(rplA), rpl2(rplB), rpl3(rplC), rpl4(rplD), rpl5(rplE), rpl6(rplF), rpl9(rplI), rpl10(rplJ), rpl11(rplK), rpl12(rplL), rpl13(rplM), rpl14(rplN), rpl15(rplO), rpl16(rplP), rpl17(rplQ), rpl18(rplR), rpl19(rplS), rpl20(rplT), rpl21(rplU,rpl21_1), rpl22(rplV), rpl23(rplW), rpl24(rplX), rpl25(rplY), rpl27(rpmA), rpl28(rpmB), rpl29(rpmC), rpl30(rpmD), rpl31(rpmE), rpl32(rpmF), rpl33(rpmG), rpl34(rpmH), rpl35(rpmI), rpl36(rpmJ), rps1(rpsA), rps2(rpsB), rps3(rpsC), rps4(rpsD), rps5(rpsE), rps6(rpsF), rps7(rpsG), rps8(rpsH), rps9(rpsI), rps10(rpsJ), rps11(rpsK), rps12(rpsL), rps13(rpsM), rps14(rpsN), rps15(rpsO), rps16(rpsP), rps17(rpsQ), rps18(rpsR), rps19(rpsS), rps20(rpsT), psbA(psbA1,psbA_1), rpoB(rpoB-1,rpoB-2), ccsA(ycf5), ccs1(ycf44), cemA(ycf10), hlip(ycf17), petM(ycf31), secG(ycf47), sufB(ycf24), tatC(ycf43), rbcR(ycf30)

**Species**	**Gene**	**locus_tag**	**Position**	**protein_id**
Prochlorococcus marinus	*atpB*	P9211_15571	1389417..1390883	YP_001551442.1
	atpF	P9211_15721	1403242..1403757	YP_001551457.1
	atpH	P9211_15741	1404363..1404698	YP_001551459.1
	ycf3	P9211_01461	143012..143533	YP_001550031.1
	ycf4	P9211_12321	1107849..1108400	YP_001551117.1
Synechocystis sp.	*atpE*	slr1330	1668531..1668941	NP_441408.2
Chlamydomonas reinhardtii	*rpoB*	ChreCp042	(96126..98006)	NP_958397.1
		+ChreCp041	+(98624..101092)	+NP_958398.1
