# Supplementary material for: Plastid genomes of two brown algae, Ectocarpus siliculosus and Fucus vesiculosus: further insights on the evolution of red-algal derived plastids
Source: BMC Evol Biol. 2009 Oct 16;9:253. doi: 10.1186/1471-2148-9-253 (PMC2765969; doi:10.1186/1471-2148-9-253)
Supplement: Additional file 3 — Partial multiple alignment of Tic20 and yfc60 protein homologs. Figure S2 showing partial multiple alignment of Tic20 and yfc60 protein homologs from red alga-derived plastid and apicomplexan genomes. [file 1471-2148-9-253-S3.PDF]

Figure S2.

```
                     *         *         *         *         *         *         *         *
Escp124   : -------------------EQKFQFDKPKKWFIAFNALLFQRGFSALAYYVPYLDMINLHIP-LLKDENPLVLRVFLPDF :  89
FvesORF76 : -------------------EQKFNIKKPRKWFFEFNTVILQRLLSIFAYYIPYLDVINTHLP-LIEETHPYLVRLFLPNY :  96
Vlito     : -------------FLIKKFKLKKVIKKSIRKFFKISLNLIERIGALFAYFVPLVSIYAAYVP-RLIGSYPYLNFIFPEFL : 166
Tgon      : PVRGPRASRLFASRATDAQNTPATASRRGTGFLVRVPSFVHRLAAAAMYFVPNLELLQTFLP-FLTTMLPSAAPLWTIA- : 269
Pkno      : -----KTSRTNAKIRHSHVHMSLKEKMNFTFQGESDITLLDKLIASTSYILPFIDAIQAFVM-PLVNMLPPSLHKYLF-Q : 138
Cmer      : ---------------IGIGVGVGVIRKVLKKGMKAEMTIGERMLCFGYYLLPVLECMTHCGP-DVLN--GWMKGLYKR-S :  77
Ccal      : ---------LVILISYKLYRYIPKRNNSNKVYKEDYPSIMARAISCLIYFLPLLEGIAQFGI-VCIDDHSWIRIIYKN-T :  89
Gten      : --------------FIIRQLYLHIAQSYKNHDVNDIT-IVDRLGSILPYWLPLLEGLQNFGQ-QILPDYPFNVMQIYKKT : 122
Pyez      : --------------LALQRAYKYIKLNKKINNTESKTRLSIRLVSTVPYYLPLFEGLQNFGQ-YVLPDYPVAAIPLYKKI :  84
Ehux      : --------------MNQRTLLPL---------------IIERGFPILAYSIPLAESIYFFAHRVCLYTNNISLKLLFLQT :  51

                     *         *         *         *         *         *         *         *
Escp124   : VADSIDLVQQIPFLTFVYISLGYGLFIRYKIP----------------KDRFIRYNVMYSIMLISLQGIINDMFITFIET : 153
FvesORF76 : IAESLQIIQQIPFLPFLYLMLGYGIFIRYKLP----------------KDRLVRFNIMYGIIIISFQGIIHELFLNFTK- : 159
Vlito     : RDSVDFYIRYPWVFNYIYFFGMMYGVMLFKKP----------------KPRFIRFHLVRGLMLFAFQGIPDACVKAFQS- : 229
Tgon      : -ARCLELYSRIPCASLLSFGAPYTLLIKKKELFK--------------PSYFLRHHTMTALLLSMLQYTLSMIYLKGV-- : 332
Pkno      : IEKLNQMYSSIPFLSFGTFMGLYFLFVKENRFK---------------FHYFIRYHHMQSLILSMFGYALALFYFRVFPY : 203
Cmer      : LGDLVVVYSTYPILGFMIFFMSYFLLVRGILQ----------------VRKKVRFHVSQALIIYLLTSIIGSLLNAL--P : 139
Ccal      : LAYIVVPYLESPLIGFCIFITLYLIFVRGIIQ----------------ISKFIKFHIVQALLLYLLDSVIGTVLTSL--P : 151
Gten      : LMPLVIFYVTHPTLAVIIFFILYYLFVRNKSPIP--------------DRPFIRFNVLQSILLFLINSLLGATFRAL--P : 186
Pyez      : ILPMLIFYMNHAILGLVTFFALYYVLVRNKSPIP--------------VHQLVRFNSMQSILLFLVGSLFGAVFRAF--P : 148
Ehux      : LKPIAMFYSNNLYAIFFTVCYIFAACSANRLPLSRLLPTREGKPICLNLSKFTRINVMQAILLEIVIAMIGQIWNLS--P : 129

                     *         *         *         *         *         *         *
Escp124   : -IILDSHIRATITLVSFFLWLALYIPCFIRSFAGKYDENKFIREAVEVHLGRDGPD-FIWWDKRRKGDEKK : 222
FvesORF76 : IFCFDSTDRSEAALLTFLGWVLIFIPCFLRALFGKYESNPFLREAIEVHLGRDGPD-FVWWDRGKNNKKPK : 229
Vlito     : SESLTQDQIVSTNLCLFAINLSWILPCIYQAITHTYPRSSFIRDAVEINVGRDKDDGFKWWNR-------- : 292
Tgon      : SASVTGTAHETIVLSLFITAQTLLLSSMFSALSAKYAWVPVVTEAVTMHIGEKPSDSY------------- : 390
Pkno      : SYNDTDIFNLTILYSTMSIYFGSLLIPFLSSLLGYYIEIPVISDAIKLHIGEKKKNE-------------- : 260
Cmer      : EMILMGWFGSTCLDILFILTMGSVIYASYQVWNGELTRLPLISEAAKLQVQDGEGEKK------------- : 197
Ccal      : LEIGYSFIGDRLADTLLLITFMISIYAGTDALKGQYSNIPLISEAAKMQSKSTD----------------- : 205
Gten      : IEFRMSLYGLMMCNTLFWFVLSTISYSIIKSIEGKYAKIPVISQAVRIQIDNQL----------------- : 240
Pyez      : IEFRISFLGLMVCNMMFWFVLSTITYSIVKAVQGKYSNIPVISEAVRIQISGYST---------------- : 203
Ehux      : TLLKNGLFGTFVANACLVGLVGLIVYSSFIIALGRYPRLPVISGAARVQLQGGWLN--------------- : 185
```

Figure S2. Partial multiple alignment (C-terminal end) of Tic20 and yfc60 protein homologs from red-alga derived plastid and apicomplexan genomes.

Species abbreviations and GenBank accession numbers: Escp124, *Ectocarpus siliculosus* Escp124 conserved hypothetical plastid protein (this study); FvesORF76, Fucus vesiculosus FvesORF76 conserved hypothetical plastid protein (this study); Vlito, *Vaucheria litorea* conserved hypothetical plastid protein (YP_002327553); Tgon, *Toxoplasma gondii* putative TIC20 protein (ACB58125); Pkno, *Plasmodium knowlesi* strain H conserved hypothetical protein (XP_002259450); Cmer, *Cyanidioschyzon merolae* plastid ORF197 (NP_848999); Ccal, *Cyanidium caldarium* conserved hypothetical plastid protein CycaCp071 (NP_045088); Gten, *Gracilaria tenuistipitata* conserved hypothetical plastid protein (YP_063537); Pyez, *Porphyra yezoensis* hypothetical plastid protein 203 (YP_537041); Ehux, *Emiliania huxleyi* hypothetical plastid RF60 (YP_277321). Conserved and similar residues are shaded according to their level of conservation. The boxed regions in the Escp124, Fves and Tgon represent transmembrane regions predicted using TMHMM program (available on the http://www.expasy.org server).
